# Focal adhesion kinase and Src mediate microvascular hyperpermeability caused by fibrinogen- γC- terminal fragments

**DOI:** 10.1371/journal.pone.0231739

**Published:** 2020-04-30

**Authors:** Xiaohua Guo, Rebecca A. Eitnier, Richard S. Beard, Jamie E. Meegan, Xiaoyuan Yang, Alexandra M. Aponte, Fang Wang, Peter R. Nelson, Mack H. Wu

**Affiliations:** 1 Department of Surgery, University of South Florida Morsani College of Medicine, Tampa, FL, United States of America; 2 Department of Biomolecular Research, Boise State University, Boise, ID, United States of America; 3 Department of Molecular Pharmacology and Physiology, University of South Florida Morsani College of Medicine, Tampa, FL, United States of America; 4 Department of Surgery, University of Oklahoma Health Sciences Center, Oklahoma City, OK, United States of America; University of Illinois at Chicago, UNITED STATES

## Abstract

**Objectives:**

We previously reported microvascular leakage resulting from fibrinogen-γ chain C-terminal products (γC) occurred via a RhoA-dependent mechanism. The objective of this study was to further elucidate the signaling mechanism by which γC induces endothelial hyperpermeability. Since it is known that γC binds and activates endothelial αvβ3, a transmembrane integrin receptor involved in intracellular signaling mediated by the tyrosine kinases FAK and Src, we hypothesized that γC alters endothelial barrier function by activating the FAK-Src pathway leading to junction dissociation and RhoA driven cytoskeletal stress-fiber formation.

**Methods and results:**

Using intravital microscopy of rat mesenteric microvessels, we show increased extravasation of plasma protein (albumin) resulting from γC administration. In addition, capillary fluid filtration coefficient (K_fc_) indicated γC-induced elevated lung vascular permeability. Furthermore, γC decreased transendothelial barrier resistance in a time-dependent and dose-related fashion in cultured rat lung microvascular endothelial cells (RLMVECs), accompanied by increased FAK/Src phosphorylation detection by western blot. Experiments with pharmacological inhibition or gene silencing of FAK showed significantly reduced γC-induced albumin and fluid leakage across microvessels, stress-fiber formation, VE-cadherin tyrosine phosphorylation, and improved γC-induced endothelial barrier dysfunction, indicating the involvement of FAK in γC mediated hyperpermeability. Comparable results were found when Src was targeted in a similar manner, however inhibition of FAK prevented Src activation, suggesting that FAK is upstream of Src in γC-mediated hyperpermeability. In addition, γC-induced cytoskeletal stress-fiber formation was attenuated during inhibition or silencing of these tyrosine kinases, concomitantly with RhoA inhibition.

**Conclusion:**

The FAK-Src pathway contributes to γC-induced microvascular barrier dysfunction, junction protein phosphorylation and disorganization in a manner that involves RhoA and stress-fiber formation.

## Introduction

When severe injury results in bleeding, fibrinogen, a soluble protein consisting of α, β and γ polypeptide pairs, is converted at the wound into fibrin by thrombin [1]. The proteolysis of fibrin is coupled with its breakdown into fibrin degradation products (FDPs), which includes a D-dimer and soluble C-termini of the α, β and γ chains [1]. Elevated plasma levels of FDPs have been documented in numerous pathological conditions, such as congestive heart failure [2], ischemic strokes [3], and myocardial infarctions [4]. Of all the soluble fibrinogen monomers, the C-terminus of the γ chain is of specific interest due to its reactivity imparted by a calcium binding site, polymerization pocket and cross-binding site. This reactive region allows for surface receptor binding and stimulates fibrin cross-linking [5]. Our previous study identified the C-terminal fragment of fibrinogen gamma chain (γC) as a mediator of microvascular leakage via association with αvβ3 integrin receptor in RhoA-dependent pathway [5], which suggested the participation of the fibrinolysis pathway in other cellular functions besides coagulation. However, the mechanism behind fibrinogen γC microvascular hyperpermeability is not fully understood.

Endothelial cells line the internal vascular surface and in conjunction with the underlying extracellular matrix (ECM) creates an important interface responsible for maintaining vascular barrier function [6, 7]. The integrity of this barrier is largely dependent on junction proteins, which are connected to the F-actin cytoskeleton via linker proteins [6, 7]. Proinflammatory mediators, including interlukin-1 (IL-1), tumor necrosis factor (TNF), vascular endothelial growth factor (VEGF), and activated neutrophils are capable of causing endothelial barrier dysfunction [8, 9]. The underlying mechanism involves cytoskeleton contraction, adherens junction (AJ) dissociation, and focal adhesion disruption [9]. Impaired barrier function results in microvascular leakage and edema, which are hallmark events in the progression of severe trauma, sepsis, multiple organ failure, and other inflammatory disease conditions [6, 9].

Tethering of the endothelial monolayer to the ECM is mediated by focal adhesion complexes, which are regulated by various signaling molecules and play a critical role in mediating adhesion, contraction and permeability [6, 10, 11]. Within this dynamic cellular environment, focal adhesion kinase (FAK) catalyzes various downstream reactions leading to focal adhesion assembly and integrin linkage allowing the endothelial monolayer to attach to the extracellular matrix [11–13]. FAK is mainly regulated through tyrosine phosphorylation at residues Y925, Y397 and Y576/577 [12]. Activation of these sites leads to focal adhesion formation, integrin binding, cell contraction, intercellular gap formation and consequential microvascular barrier dysfunction [12–14]. Numerous inflammatory mediators have been reported to activate FAK and cause increased transendothelial permeability [10, 15]. Our laboratory and others have shown that inhibition of FAK attenuates vascular hyperpermeability in response to VEGF [8, 14], activated neutrophils [13], and advanced glycation end products (AGEs) [16].The interaction between FAK and other tyrosine kinases, such as c-Src, a non-receptor tyrosine kinase belonging to the Src family kinases (SFKs), has been well established over the past decades [12, 15, 17, 18]. Studies have shown that Src induces vascular permeability through focal adhesion complex interactions and phosphorylation of Vascular Endothelial (VE)-cadherin, which results in dissociation of cadherin-catenin-actin AJ complexes [16]. Previous studies have demonstrated that Src inhibition attenuates TNFα-induced pulmonary vascular hyperpermeability via restoring VE-cadherin integrity [19]. Blocking the Src pathway can also reduce β-catenin phosphorylation and neutrophil-induced vascular hyperpermeability [20].

It is well documented that FAK and Src activity are heavily associated with the RhoA pathway [12, 21]. Activation of FAK and Src increase phosphorylation of cytoskeletal proteins, including p190RhoGAP, a key regulator of Rho activity [21, 22]. Activation of small GTPases RhoA play a central role in regulating actin dynamics, and RhoA is directly responsible for F-actin stress fiber formation [23]. The close correlation between F-actin stress fiber formation and loss of endothelial monolayer integrity in response to hyperpermeability factors has been reported [24, 25].

Despite the increasing evidence that FDPs contribute to inflammation [3–5], physiological mechanism of the disease development remains to be decided due to complexity of fibrinogen’s multi-domain structure and its diverse cellular targets. In our previous study, we notated γC stimulated integrin-mediated barrier dysfunction by targeting αvβ3 [5]. Considering that the role of FAK and Src are well documented in vascular leakage and the development of inflammatory diseases [12, 13, 26, 27], and its association with αvβ3 integrin signaling was reported [5], FAK and Src have emerged as potential mediators to transmit the γC barrier response. In the current study, we sought to determine if FAK and/or Src activity drives γC microvascular leakage. Assessing the physiological impact of targeting these kinases may provide new information for developing therapeutic strategies in improving severe trauma-induced vascular hyperpermeability.

## Materials and methods

### Reagents and supplies

A detailed list of reagents, supplies, and vendors is provided in S1 Table.

### Intravital microscopy

Protocol instructions were followed as previously described [5]. Rats (males weighing 200–300 g) were anesthetized with an intramuscular injection of urethane at 1.75 g/kg. Right carotid artery was cannulated and connected to a pressure transducer for continuous blood pressure monitoring by a DigiMed Blood Pressure Analyzer (Micro-Med; Lousiville, KY). The left jugular vein was cannulated for infusion of drugs and/or solutions. A midline laparotomy was performed, and a section of the proximal ileum mesentery was exteriorized over the optical stage for microscopic observation. The exteriorized mesentery was constantly superfused with 37°C lactated Ringer’s Injection USP (Baxter Healthcare; Deerfield, IL). The body temperature was maintained at 37°C using a heating pad (Fine Science Tools; North Vancouver, BC) and animals received continuous intravenous infusion of lactated Ringer’s at 0.04 mL/min/kg of body weight to replenish fluid loss during the experiment.

Rat mesenteric microcirculation was examined using a Nikon Eclipse E600FN Microscope equipped with a Photometrics Cascade 512F digital camera under a 10X working distance objective (Technical Instruments, Burlingame, CA). The diameter of resistance arterioles and postcapillary venules were measured. For measurement of plasma protein extravasation, rats were given an intravenous bolus of FITC-albumin at 100 mg/kg of body weight, followed by continuous infusion at 0.15 mg/kg/min to maintain a constant plasma tracer concentration. Mesentery preparation was equilibrated for 30 minutes to establish a steady baseline, prior to image collection. Fluorescent images were analyzed using Simple PCI version 5.3.1 software (Compix Imaging Systems; Cranberry Township, PA). Fluorescence intensity was measured from windows positioned inside (Ii) and outside (Io) a selected venule. Albumin transvascular flux was determined from the ratio of the transmural fluorescence intensity difference to the initial intravascular intensity normalized to background intensity, and calculated as 1-(Ii- Io)/Ii [28].

### Capillary filtration coefficient (K_fc_) measurement

Wild-type (WT) C57BL6 mice (weighing 25–30 g) were used for isolated perfused lung experiments and K_fc_ measurement analysis, following the protocol described [29]. The isolated-perfused mouse lung was instrumented with arterial cannula at the base of the pulmonary artery and left atrial cannula, and ventilated (186 breaths/min) and perfused with 3% albumin-krebs 37°C solution at a constant rate of 2 mL/min for 20 minutes to equilibrate. All preparations were mechanically ventilated and arterial and venous pressures were monitored continuously throughout the experiment. Perfusion pressure of lung preparations varied from 7 to 12 cm^3^ H_2_O, and remained within ~1 cm^3^ H_2_O of the initial value over the course of an experiment.

The capillary filtration coefficient (*K*_fc_) was measured to determine pulmonary microvascular permeability to liquid, as described [29]. After perfused lungs were equilibrated for 20 minutes, γC was infused for 5 minutes via cannulated pulmonary artery at a rate of 0.2 mL/min, at a final concentration of 10 μg/mL with or without pre-perfused FAK inhibitor (PF-573228, 1 μM), or Src inhibitor (PP2, 10 μM) for 30 minutes. Lung wet weight changed in a ramplike fashion, emulating net fluid extravasation. At the end of the experiment, lungs were dissected free of non-pulmonary tissue, and lung dry weight was determined in order to calculate K_fc_, which was measured as slope of recorded weight change normalized to pressure change and lung dry weight.

### Animals

The protocols using rats (Sprague-Dawley) and mice (C57BL6 wild type) were approved by the Institutional Animal Care and Use Committee (IACUC) at the University of South Florida and were in accordance with the National Institutes of Health Guides for the Care and Use of Laboratory Animals and the guidelines of the Animal Welfare Act published by the US National Institutes of Health (NIH Publication ON. 85–23, revised 1996).

### Cell culture

Rat Lung Microvascular Endothelial Cells (RLMVECs) were cultured in gelatin-coated 100 mm dishes, 6-well plates, ECIS assays or chambered glass microwells. Cells were incubated in a 5% CO_2_ humidified incubator at 37°C to reach confluence 2–3 days prior to experiments.

### Recombinant fibrinogen C-terminal domains

Recombinant γC fragment (residue 151–411, 30 kD) was produced as previously described [5]. Its cDNA was cloned into the pET21a vector and overexpressed in E. coli. Then, the recombinant protein was renatured through urea step gradient and refolded by dialysis. The refolding buffer was exchanged for PBS, followed by detoxification, in which the final product was suspended in endotoxin-free PBS.

### Gene silencing

Transfection with FAK or Src siRNA duplexes (see Supplementary Materials and Methods) were conducted by electroporation using Nucleofector^™^ II Device (Lonza, Amaxa^™^ Biosystems; Cologne, Germany) as previously described [30]. Briefly, cells were trypsinized, pelleted, and resuspended in Basic Endothelial Nucleofector Solution containing siRNA duplexes [0.4 μM]. Immediately after electroporation with program T-011, cell suspension was mixed with 500 μL of pre-warmed MCDB-131 complete medium and seeded on 100 mm dishes, 6-well plates, or ECIS arrays. Endothelial cells were treated as designed 48 hours post-transfection.

### Transendothelial Electrical Resistance (TER)

Endothelial barrier function was determined by measuring transendothelial resistance using an electric cell-substrate impedance sensing (ECIS; Z theta; Applied Biophysics, Inc.) system [31]. RLMVECs were subcultured onto the ECIS electrode arrays at 1 × 10^5^ cell/cm^2^, and allowed to reach two-days post-confluence. A 1 V, 4,000 Hz alternating signal was connected serially through a 1 MΩ resistor between the two electrodes to supply a constant current of 1 μA. The in-phase voltage (proportional to resistance) and the out-of-phase voltage (proportional to capacitive resistance) were measured and analyzed with ECIS_Core software (v1.2.210.1 Mac; Applied BioPhysics, Inc.). ECIS tracings of TER are presented as either resistance (Ohms) or normalized change in resistance, i.e. normalized to baseline resistance at time of stimulus with gamma C peptides.

### Rho activity assay

Rho activation in endothelial cells was assessed using a Rho-GTP pulldown assay kit as previously described [25]. Briefly, confluent endothelial cells were serum deprived in EBM medium with 0.5% FBS for 4 hours prior to treatment. Cells were washed twice with TBS and lysed in Mg^2+^ lysis/wash buffer [MLB: 25 mM HEPES (pH 7.5), 150 mM NaCl, 1% Igepal CA-630, 10 mM MgCl_2_, 1 mM EDTA, 10% glycerol, 10 μg/mL leupeptin, 25 mM NaF, and 1 mM vanadate] with protease/phosphatase inhibitor cocktail. Cell lysates were incubated with Rhotekin-Rho-binding domain on a rotator at 4°C for 1 hour. The beads were washed 3 times with MLB and incubated in 2×SDS sample buffer at 95°C for 5 minutes to elute protein. Active RhoA was then separated by SDS-PAGE and detected by immunoblotting.

### Immunocytochemistry

RLMVECs were cultured on gelatin-coated glass bottom microwells to confluency. Immunocytochemistry was performed per standard protocols and as we have previously described [32]. Briefly, after appropriate treatments, cells were fixed with 4% PFA and permeabilized with 10% donkey serum containing 0.05% Triton X-100. After blocking, cells were immunostained with VE-cadherin (VE-cad) and β-catenin (β-cat) antibodies at 4°C overnight and appropriate fluorescent conjugated antibodies for 1 hour at RT. F-actin was stained by Alexa Fluor 647-phalloidin (2,000 U/mL, 20 minutes) at room temperature. Slides were mounted in Vectashield containing DAPI and imaged with a FLUOVIEW FV1000 confocal laser scanning microscope (Olympus) using a 60X objective (UPLFLN 60X OI NA:1.25). Confocal micrograph stacks were analyzed and processed with Imaris (Bitplane) or NIH ImageJ. As an indicator of adherens junction integrity (Fig 6), intensity analyses of VE-cadherin and β-catenin were measured at cell-cell junctions and quantified in three different ways: 1) VE-cad/β-cat colocalization—Pearson’s correlation coefficient was derived from their scatterplots (Fig 6B); 2) the total surface area of gaps between cells within the RLMVEC monolayer (Fig 6D); or 3) as disruptions in pixel-to-pixel tracings of positive VE-cadherin staining at cell-cell contacts (Fig 6E). As an indicator of stress fiber formation, phalloidin staining was quantified by either total intensity within the micrograph (Fig 7B); or intensity of cytoplasmic and perinuclear ROI’s (Fig 7C).

### Western blotting

RLMVECs were grown to confluence in MCDB-131 complete medium, treated as designed, and lysed in lysis buffer [20 mM Tris (pH 7.4), 2.5 mM EDTA, 1% Triton X-100, 1% deoxycholic acid, 0.1% SDS, 100 mM NaCl, 10 mM NaF, 1 mM Na3VO4] containing protease/phosphatase inhibitor cocktail. Lysates were collected and clarified by centrifugation at 13,000 *g* for 20 minutes, prior to SDS-PAGE, and transferring proteins to PVDF membranes. Blots were then blocked using 5% BSA in TBST (TBS containing 0.5% Tween20) for 1 hour, followed by overnight incubation at 4°C on a rocker in 1:1000 dilution of the primary antibody of interest. The following day, membranes were washed three times for 5 minutes each with TBST, the membrane was incubated with a corresponding horseradish peroxidase (HRP) conjugated secondary antibody for 1 hour at room temperature. Membranes were washed after secondary incubation three times for 5 minutes each with TBST, and protein bands were visualized by chemiluminescence.

### Immunoprecipitation

RLMVECs were washed once with PBS and lysed in IP lysis buffer (20 mM Tris-Cl (pH 7.5), 150 mM NaCl, 1% Triton X-100, 1 mM EDTA, 1 mM EGTA, 2.5 mM sodium pyrophosphate, 1mM β-glycerol phosphate, 1 mM Na3VO4) containing protease/phosphatase inhibitor cocktail. The lysates were clarified by centrifugation at 14,000 rpm for 10 minutes, and supernatants were incubated in 1 μg of corresponding antibody with gentle rocking overnight at 4°C. The following morning, samples were incubated with protein A-Sepharose suspension at 4°C for 1 hour. The beads were washed 3 times in the IP lysis buffer, heated in 2x SDS sample buffer at 95°C for 5 minutes, and resolved on SDS-PAGE followed by immunoblotting.

### Statistical analyses

All statistics were performed using GraphPad Prism (version 6.0f). Unless otherwise noted, all data represent the mean and s.e.m. Unpaired, two-tailed, T-tests were used for pairwise comparisons and One-way ANOVA with Tukey’s post-hoc analysis was used for comparisons of 3 or more groups. α was set at 0.05 *a priori* for statistical significance.

## Results

### γC-induced microvascular leakage and endothelial barrier dysfunction

**Fig 1:**
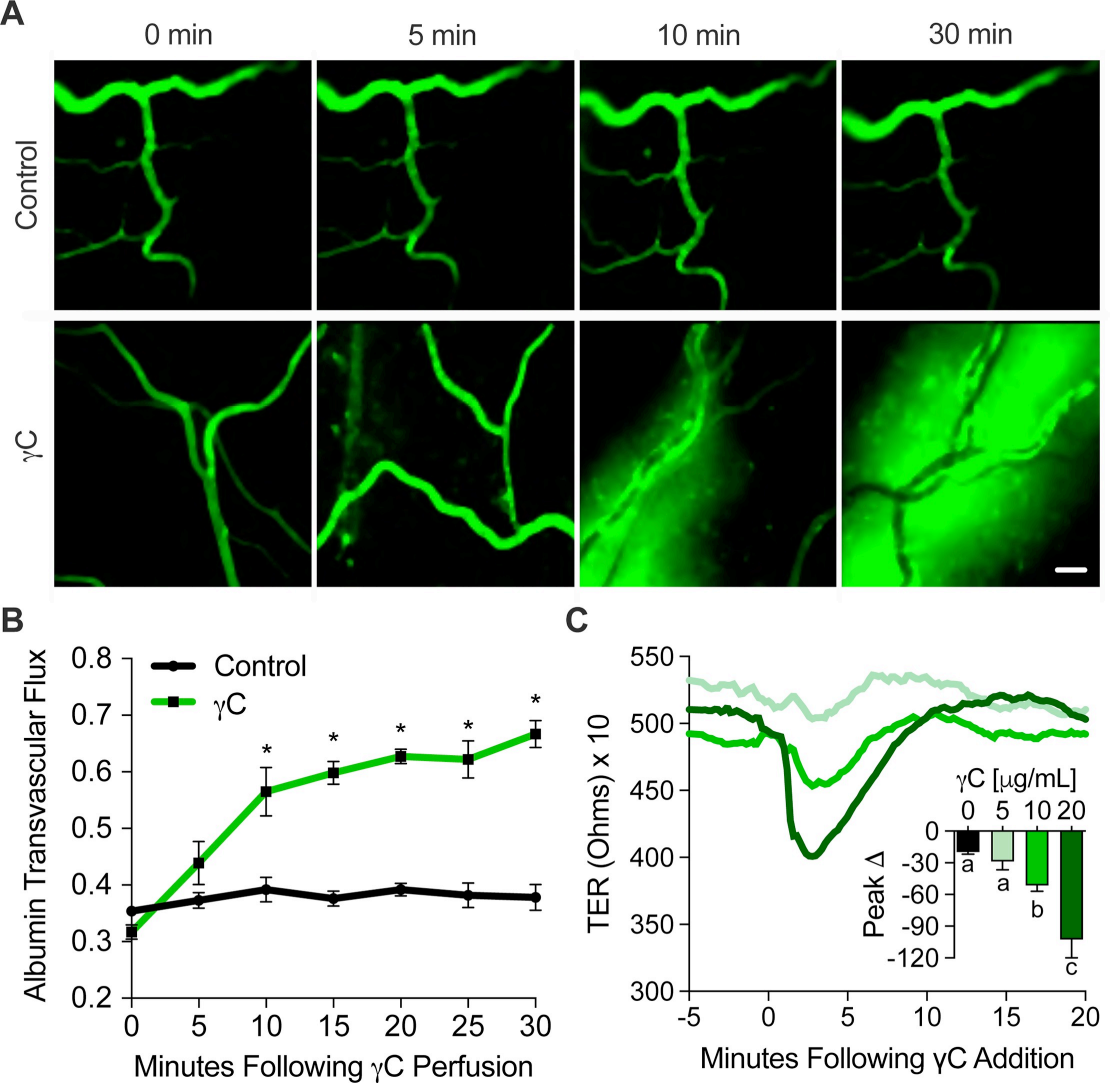
γC induces microvascular hyperpermeability and endothelial barrier dysfunction. Rat proximal ileum mesenteric microcirculation was exteriorized on microscopic platform. FITC-Albumin was allowed to equilibrate through circulation prior to treatment with γC (10 μg/mL). (A) Leakage of γC was observed at 5 minutes and continued throughout the recorded duration of 30 minutes, representative images taken at 0, 5, 10, and 30 minutes are shown, scale bar = 40 μm. (B) Quantitative analysis showing increased albumin transvascular flux across mesenteric microvessels with γC treatment vs. control. Data represent means ± s.e.m. * indicates p < 0.05 vs. timepoint 0, (n = 4). Primary rat lung microvascular endothelial cell (RLMVEC) monolayers grown in ECIS arrays and treated with γC dosages of 5, 10, and 20 μg/mL while transendothelial resistance (TER) measurements were recorded (C) Representative TER tracings are shown and peak TER reduction representing barrier dysfunction was calculated by subtracting the lowest resistance following γC addition to media. Embedded bar graphs represent the mean peak change and s.e.m. Groups with the same letter are not significant from each other (p > 0.05), (n = 4).

We began our study by testing the effect of γC on cultured RLMVECs. Transendothelial electrical resistance (TER) was obtained using ECIS arrays, after RLMVECs were plated and grown to 2-day post-confluence. We found that °C induces barrier dysfunction in a dose-related manner (Fig 1C). Using 10 μg/mL as an effective dosage, the impact of °C on rat mesenteric microvascular leakage was tested. Using intravital microscopy, FITC-albumin flux was observed after perfusion of °C or vehicle control into mesenteric microcirculation. °C treatment increased microvascular leakage of albumin throughout the time intervals of 5, 10 and 30 minutes (Fig 1A). The albumin transvascular flux was recorded as control vs. °C treatment (Fig 1B) for the given time intervals. *In vivo* and *in vitro* experiments denoted the harmful effect of γC on the endothelial barrier.

### FAK inhibition attenuates γC-induced microvascular leakage and barrier dysfunction

**Fig 2:**
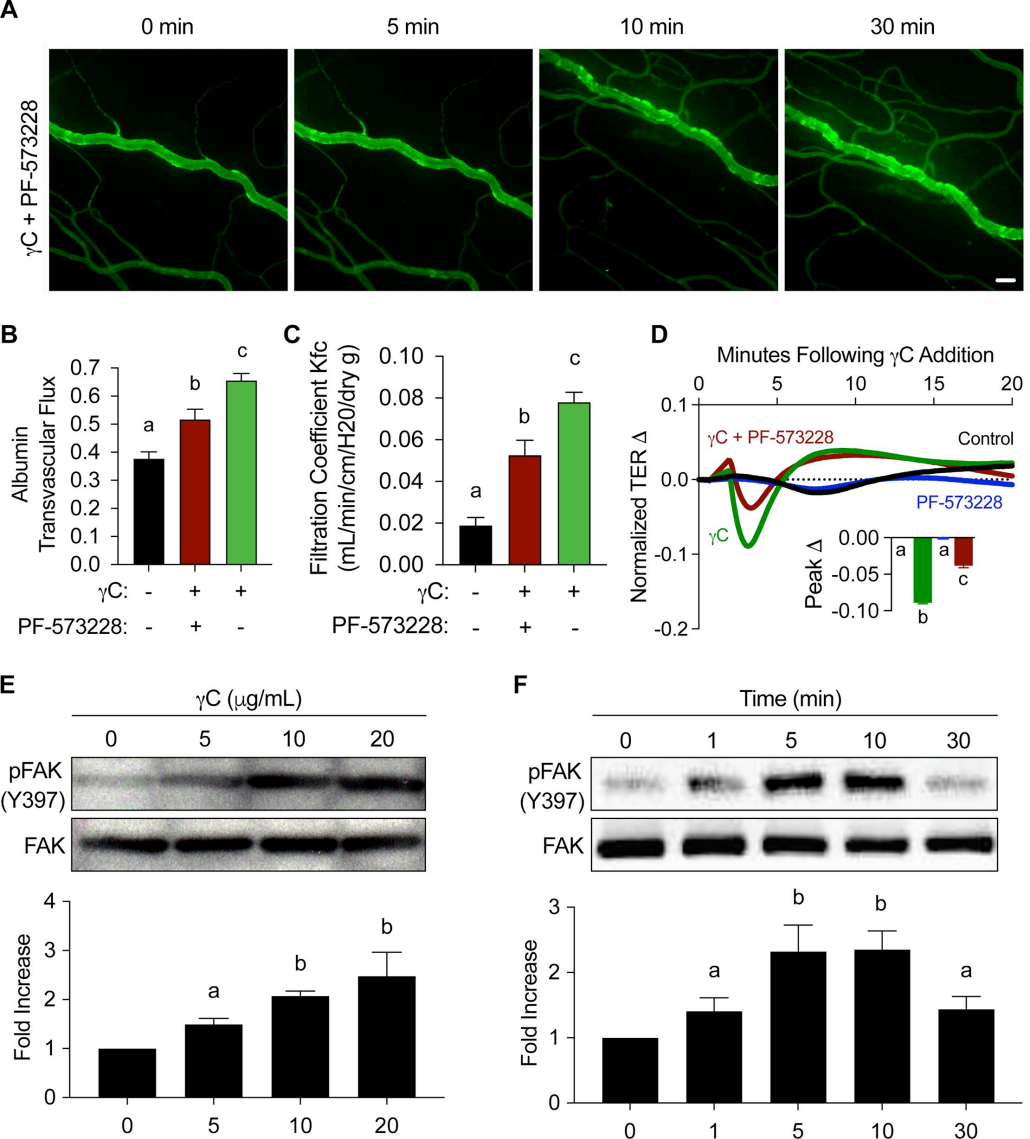
FAK inhibition reduces γC-induced microvascular hyperpermeability and barrier dysfunction. Rat proximal ileum mesenteric microvessels were exteriorized on microscopic platform, equilibrated with FITC-Albumin, pretreated with FAK inhibitor (PF573228, 1 μM) for 30 minutes prior to 10 μg/mL γC treatment. A) Visualization of rat ileum microvessels treated with FAK inhibitor prior to γC treatment show reduced microvascular leakage at 5, 10, and 30 minutes when compared to γC only treatment (Fig 1A). Images are representative of four individual experiments. Scale bar = 40 μm. B) Quantitative analysis of albumin transvascular flux at 10 min was compared with control and γC only treatment values obtained from Fig 1A, (n = 4). C) While perfusing isolated mouse lung preparations, capillary filtration coefficients (Kfc) were measured and demonstrated elevated fluid filtration in response to γC which was reduced when pretreated with PF573228, (n = 4). D) RLMVEC monolayers in ECIS arrays were treated with PF573228 (1 μM) prior to addition of γC (10 μg/mL). Representative ECIS tracing of normalized change in TER are shown with a bar graph insert of the mean peak changes, (n = 4). E-F) RLMVECs were treated with γC dosages of 0, 5, 10, and 20 μg/mL (E) and 10 μg/mL at indicated time points of 0, 1, 5, 20, 30 minutes (F) followed by detection of total FAK and phospho-FAK Y397 specific antibodies. Blots and average optical densities are representative of three independent experiments (n = 3). Bar graphs represent the means ±s.e.m. Groups with the same letter are not significant from each other (p > 0.05).

Since FAK has be implicated in integrin-mediated barrier dysfunction [10] and previous experiments denote the binding of γC to integrin αvβ3 [5], we sought to deduce the role of FAK in γC-induced microvascular hyperpermeability. Rat ileum mesenteric microvessels were pretreated with pharmacological inhibitor (PF573228 at 1 μM for 30 minutes) prior to γC or vehicle control treatment and FITC-albumin flux was measured via intravital microscopy (Fig 2A and 2B). The capillary filtration coefficient (K_fc_) was measured via perfusion of a cannulated mouse pulmonary artery with γC (10 μg/mL) with or without pre-treatment of FAK inhibitor (PF573228, 1 μM, 30 minutes). Data show that FAK inhibition reduces γC-induced microvascular hyperpermeability (Fig 2C). After growing RLMVECs on ECIS arrays to 2-day post-confluence, TER was measured and showed that treatment with PF573228 attenuates γC-induced barrier dysfunction (Fig 2D). Immunoblotting shows that γC induces phosphorylation of FAK Y397 in a dose-related manner (Fig 2E). Using γC dose of 10 μg/mL, since it caused a significant drop in barrier function using TER measurement (Fig 1C), we demonstrated an increase in phosphorylation of FAK (Y397) that was temporally consistent with γC-induced barrier dysfunction (Fig 2F). All western blot analysis showed no noticeable difference in total FAK expression. Taken together, these data suggest that γC-induced endothelial barrier dysfunction and vascular hyperpermeability are dependent on FAK activity.

### Src inhibition attenuates γC-induced microvascular leakage and barrier dysfunction

**Fig 3:**
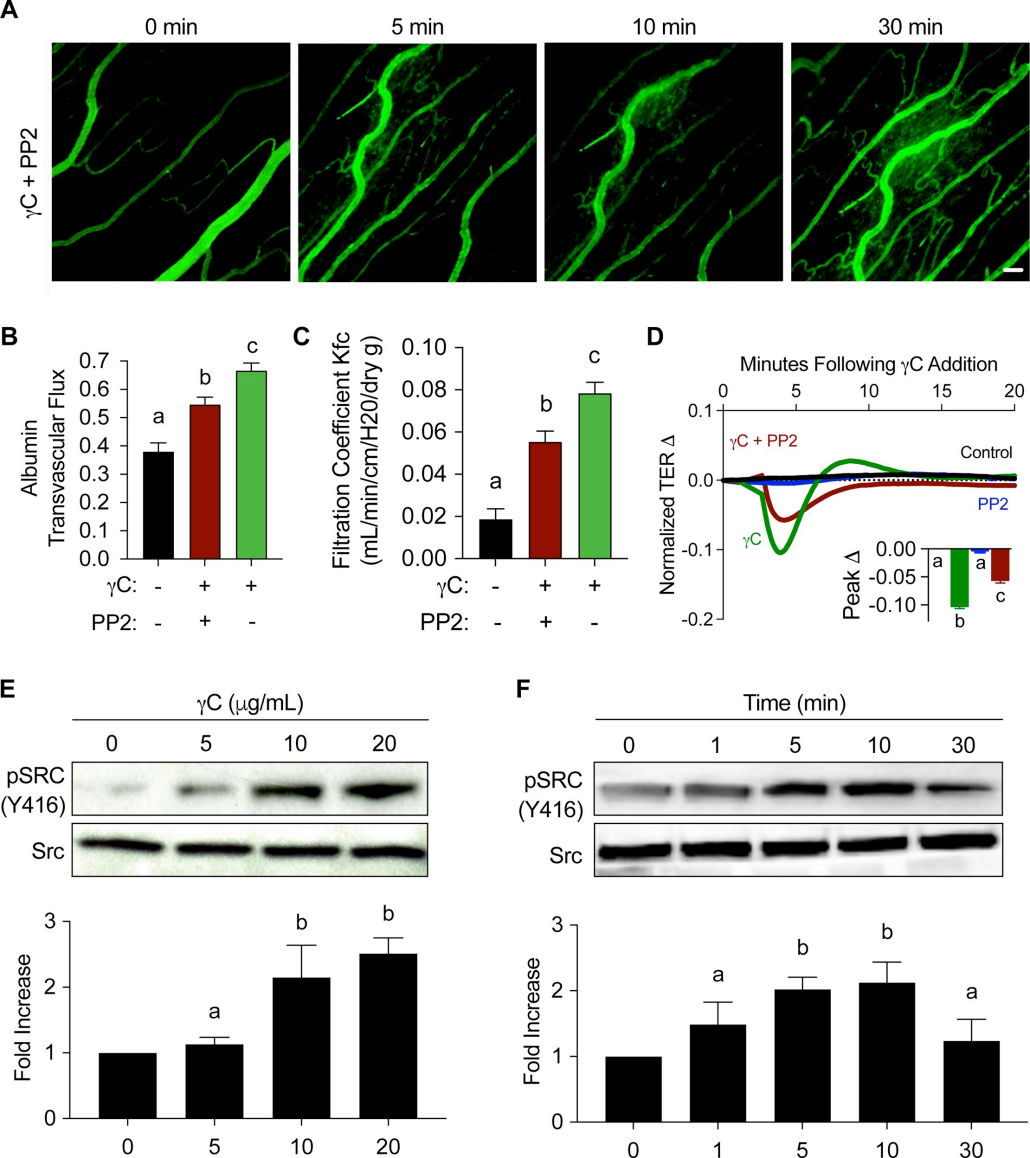
Src inhibition reduces γC-induced microvascular hyperpermeability and barrier dysfunction. Rat proximal ileum mesenteric microvessels were exteriorized on microscopic platform, equilibrated with FITC-Albumin, pretreated with Src inhibitor (PP2, 10 μM) for 30 minutes prior to γC (10 μg/mL). A) Visualization of rat ileum microvessels treated with PP2 prior to γC treatment show reduced microvascular leakage at 5, 10, and 30 minutes when compared to Fig 1A γC only treatment. Images are representative of four individual experiments. B) Quantitative analysis of albumin transvascular flux (10 mins) shown in Fig 3A compared with control and γC only treatment values obtained from Fig 1A, demonstrate that PP2 attenuates γC effect on albumin transvascular flux by approximately 50% (n = 4). C) Using isolated perfused mouse lung preparations, demonstrates elevated capillary filtration coefficient (K_fc_) in response to γC and an approximate 35% reduction when pretreated with PP2, (n = 4). D) RLMVECs were grown on ECIS arrays, pretreated with PP2 (10 μM) and then γC (10ug/mL). Peak TER reduction representing barrier dysfunction was calculated showing an approximate 50% attenuation of γC response with Src inhibition (n = 4). E-F) RLMVECs were treated with γC dosages of 0, 5, 10, and 20 μg/mL and 10 μg/mL at indicated time points of 0, 1, 5, 20, 30 minutes followed by detection of total Src and Phospho-Src Y416 specific antibodies. γC-induced phosphorylation of Src (Y416) in a dose-related manner and peak Src phosphorylation (Y416) was seen at 10 minutes (E) Blots and average optical densities are representative of three independent experiments, (n = 3). Bar graphs represent the means ±s.e.m. Groups with the same letter are not significant from each other (p > 0.05).

Once activated through phosphorylation of Y416, Src can phosphorylate many proteins involved in regulation of the endothelial barrier [20, 26, 33], therefore we asked whether Src activation was involved in γC-elicited microvascular leakage. Analysis of albumin transvascular flux shows quantification of γC-induced microvascular leakage is attenuated in response to Src inhibition with PP2 (Fig 3A and 3B). The capillary filtration coefficient (K_fc_) was measured via perfusion of a cannulated mouse pulmonary artery with γC (10 μg/mL) with or without pre-treatment of Src inhibitor (PP2, 10 μM, 30 minutes). Results show that Src inhibition reduces γC-induced microvascular hyperpermeability (Fig 3C). *In vitro* studies show Src pharmacological inhibitor (PP2) attenuated γC barrier dysfunction, using TER as a measurement of endothelial barrier function (Fig 3D). Immunoblotting shows that γC increases phosphorylation of Src (Y416) in a dose-related manner (Fig 3E). Using γC [10 μg/mL] treatment, we determined that γC-induced Src phosphorylation at Y416 is temporally consistent with FAK phosphorylation and barrier dysfunction (Fig 3F). All western blot analyses showed no noticeable difference in total Src expression. Taken together, these data imply that Src activation was necessary for γC-elicited barrier dysfunction and microvascular hyperpermeability.

### γC-induced Src activation requires FAK

**Fig 4:**
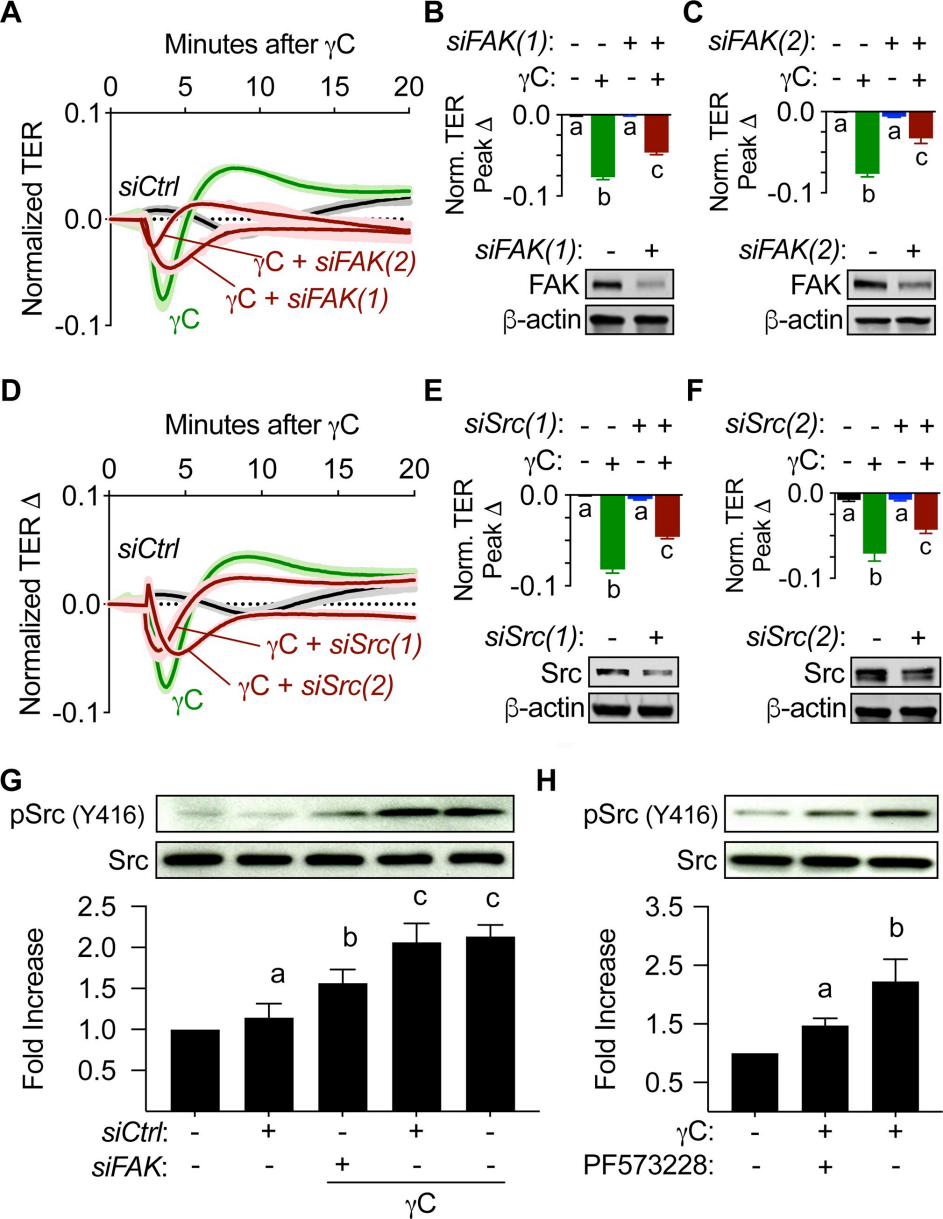
siRNA knockdown of FAK and Src reduces γC-induced barrier dysfunction and FAK inhibition reduces phosphorylation of Src (Y416). RLMVECs were grown on ECIS arrays and transfected as indicated with two independent pools of siRNA duplexes targeting *FAK*, *siFAK(1)* and *siFAK(2)*, or *Src*, *siSrc(1)* and *siSrc(2)*, prior to treatment with γC (10 μg/mL). A) ECIS tracings comparing the effect of FAK knockdown with *siFAK(1)* and *siFAK*(*2)* on γC-mediated barrier dysfunction (n = 4). B,C) Peak TER reduction in response to γC (top) was attenuated by FAK knockdown with both (B) *siFAK(1)* and (C) *siFAK(2)* siRNA duplex pools (n = 4). Representative blots (bottom) confirm knockdown. D-F) similar effects of gene silencing with *siSrc(1)* and *siSrc(2*) (n = 4) on ECIS tracings, peak TER and representative blots (bottom) to confirm knockdown. G) RLMVECs treated with *siFAK(1)* displayed a significant reduction of γC-induced Src phosphorylation (Y416) relative to the cells treated with control siRNA, (n = 4). H) Similarly, the cells treated with FAK inhibitor (PF573228, 1 μM) for 30 minutes, prior to 10-minute γC (10 μg/mL) treatment, reduced γC-induced Src phosphorylation. TER calculations, blots and average optical densities are representative of four independent experiments, (n = 4). ECIS tracings and bar graphs represent the mean ± s.e.m. Groups with the same letter are not significant from each other (p > 0.05).

To confirm that gene silencing of FAK and Src warranted similar effects on γC induced barrier dysfunction as pharmacological inhibition (shown in Figs 2 and 3), we transfected RLMVECs monolayers with two sets of pooled siRNA sequences for both FAK and Src to ensure the effectiveness of FAK or Src knockdown before the treatment with γC (10 μg/mL) and quantification of the peak TER reduction using ECIS assays. The information of these pooled siRNAs is detailed in Supplementary Material. Gene silencing of FAK and Src attenuated γC-induced barrier dysfunction (Fig 4A–4F). Given that FAK acts as an upstream regulator of Src [7, 34, 35], we investigated whether FAK is also an upstream regulator of Src in γC-induced barrier dysfunction. RLMVEC monolayers were transfected with FAK siRNA or pretreated with PF573228 before application of γC (10 μg/mL). Western blot analysis found that inhibition of FAK via pharmacological inhibitor PF573228 or FAK siRNA greatly attenuated γC-induced increase in Src phosphorylation of Y416 (Fig 4G and 4H). All western blot analysis showed no noticeable difference in total Src expression.

### FAK and Src activation mediate γC-mediated Adherens Junction (AJ) dissociation

**Fig 5:**
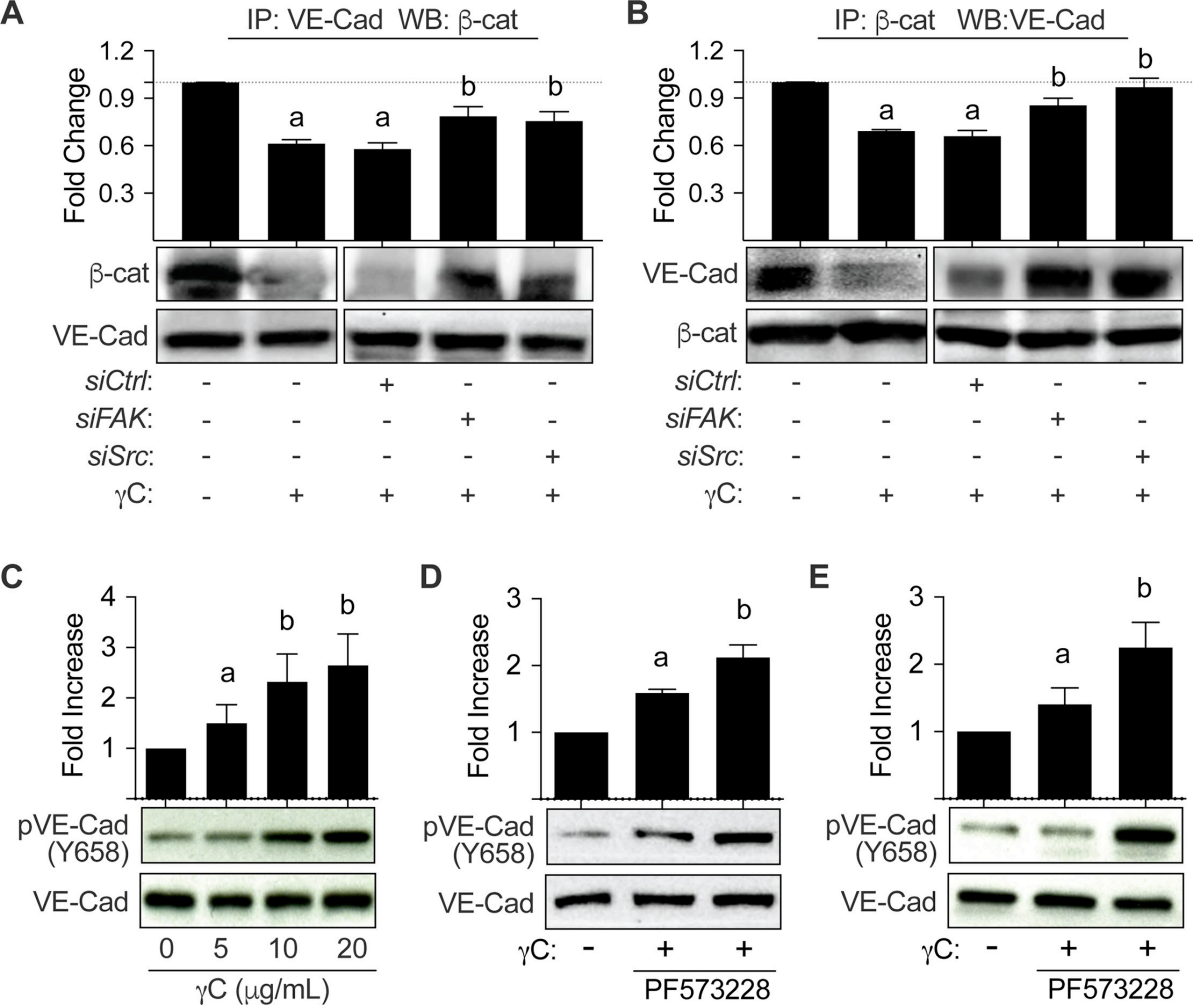
FAK and Src inhibition rescues γC induced barrier dysfunction through Adherens Junction (AJ) dissociation. RLMVECs were grown to 70% confluency, transfected with control, FAK, or Src siRNA, and then treated 48 hours post-transfection with γC (10 μg/mL) or vehicle control for 10 minutes followed by immunoprecipitation of VE-cad and blotting against β-Catenin (A) or by immunoprecipitation of β-Catenin and blotting against VE-cad (B). Densitometry analysis of these data indicates that γC resulted in a significant reduction in VE-cad and β-cat co-immunoprecipitation, which was attenuated by both FAK and Src siRNA-mediated silencing (n = 3) C) RLMVECs were treated with indicated concentration of γC for 10 minutes. Cells were lysed and analyzed for VE-cad phosphorylation (pY658). γC induced a concentration dependent increase in phosphorylation of VE-cad Y658, (n = 4). D) RLMVECs were treated with vehicle control or FAK inhibitor (PF573228, 1μM) for 30 minutes, prior to γC (10 μg/mL) for 10 minutes, and then lysed and analyzed for pVE-cad Y658. FAK inhibition reduced the increased phosphorylation of VE-Cadherin by γC, (n = 4). E) RLMVECs were treated with vehicle control or Src inhibitor (PP2, 10μM) for 30 minutes prior to γC (10 μg/mL) for 10 minutes, and then lysed and analyzed for pVE-cad Y658. Src inhibition attenuated γC-induced VE-cad (Y658) phosphorylation (n = 4). Bar graphs represent the mean ± s.e.m. Groups with the same letter are not significant from each other (p > 0.05).

In order to determine the mechanism by which the barrier is being disturbed, we sought to establish a relationship between γC-induced barrier dysfunction and junctional proteins. As indicated by immunoprecipitation of both VE-cadherin (VE-cad) and β-catenin (β-cat), we found that γC caused VE-cad/β-cat uncoupling which was attenuated by inhibition of FAK and Src (Fig 5A and 5B). Additionally, γC was found to induce VE-cad phosphorylation of residue Y658 in a dose-related manner (Fig 5C). Pharmacological inhibition of FAK and/or Src using PF573228 and PP2, respectively, reduced γC-induced VE-Cadherin phosphorylation of Y658 (Fig 5D and 5E). Furthermore, the cultured ECs with reduced level of FAK by siRNA treatment displayed attenuated phosphorylation of VE-Cadherin Y658 (data not shown).

**Fig 6:**
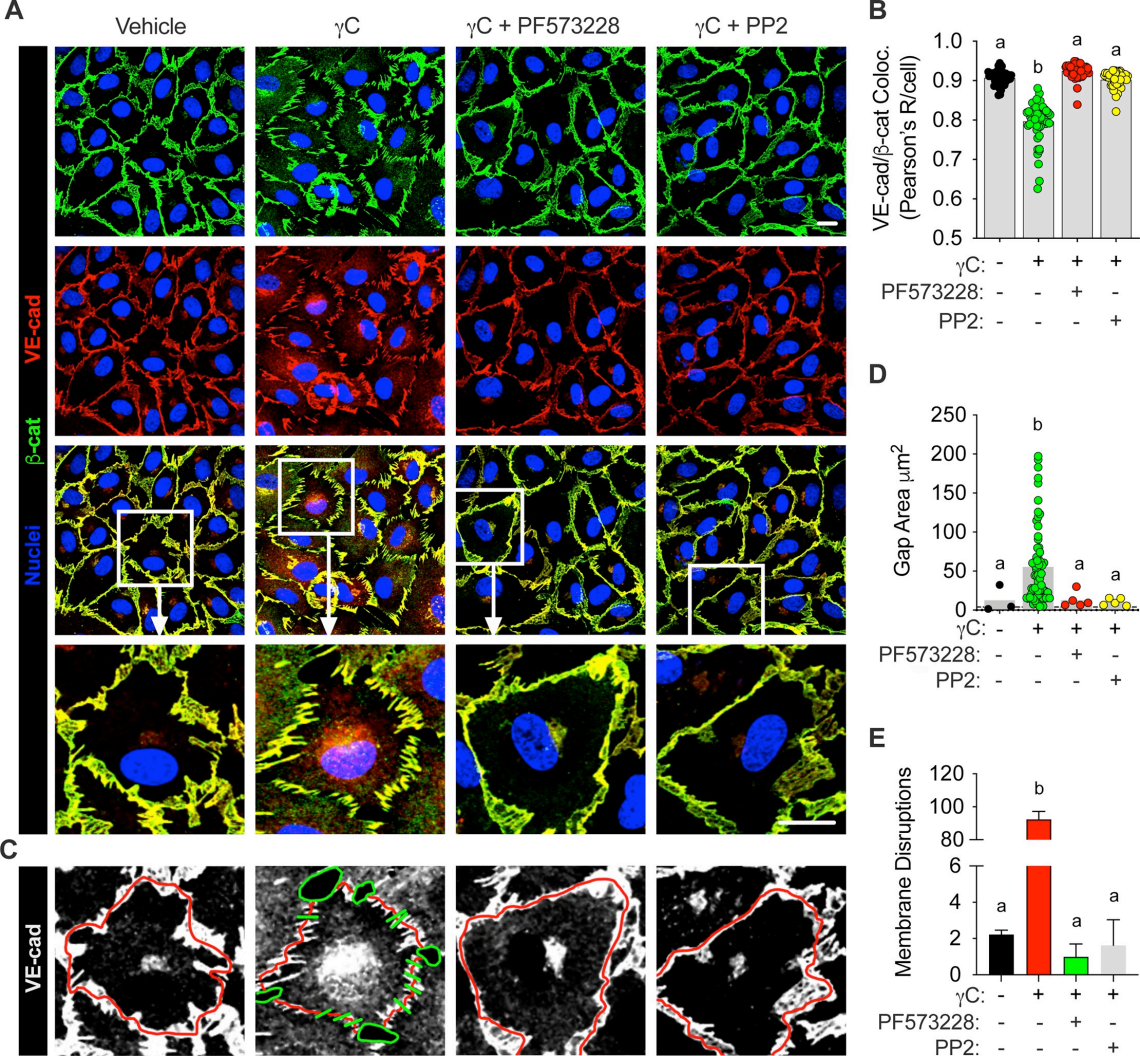
FAK and Src inhibition reduces γC-induced junctional protein disorganization. RLMVECs were grown to confluence on glass bottom microwells and pre-treated with vehicle control, FAK (PF573228, 1μM), or Src inhibitor (PP2, 10 μM), followed by γC (10 μg/mL) treatment for 10 minutes prior to immunofluorescence staining for VE-Cadherin and β-Catenin, and imaging with confocal microscopy. A) Representative confocal micrographs of RLMVECs stained for VE-cad and β-cat indicated γC-induced AJ rearrangement and dissociation, which were reversed with FAK and/or Src inhibition. B) Colocalization analysis was used as an indicator of VE-cad and β-cat coupling at cell-cell borders and Pearson’s correlation (R) coefficients were quantified per cell across three independent experiments. Results are presented as scatter dot plots with bars, where individual dots represent Pearson’s R/cell and bars indicate the mean across experiments. C-E) Using maximum projections for VE-cad on the same images the total surface area of gap formations (D) and the number of discontinuous labeling at cell-cell borders (E) were quantified. C) The representative grey scale images of VE-cad are shown to demonstrate how pixel-to-pixel tracings of positive VE-cad (red lines) were done, and at areas where the tracing was negative for VE-cad a perpendicular green line was used to indicate a ‘disruption’. The representative gaps are shown by continuous green lines encircling the borders of a gap area. The scatter dot plots and bars indicate the number of cells measured in micrographs over 3 independent experiments. Bars graphs represent the mean ± s.e.m. (n = 3). Groups with the same letter are not significant from each other (p > 0.05).

Immunocytochemistry experiments further demonstrate the effect of γC on junctional protein uncoupling and gap formations in RLMVECs (Fig 6). Using immunofluorescence confocal microscopy showed that γC induces junctional protein dissociation, as indicated by decreased VE-cad and β-cat colocalization that was attenuated by FAK or Src pharmacological inhibition (Fig 6A and 6B). Additionally, 3D-representation of the VE-cad junctional staining (Fig 6C and 6D) demonstrates that Src and FAK inhibition rescues γC-induced gap formations at cell-cell borders, which was quantified as total surface area of gaps (Fig 6D), or non-continuous staining around cell borders (Fig 6E).

### FAK and Src in RhoA-dependent γC-induced endothelial barrier dysfunction

**Fig 7:**
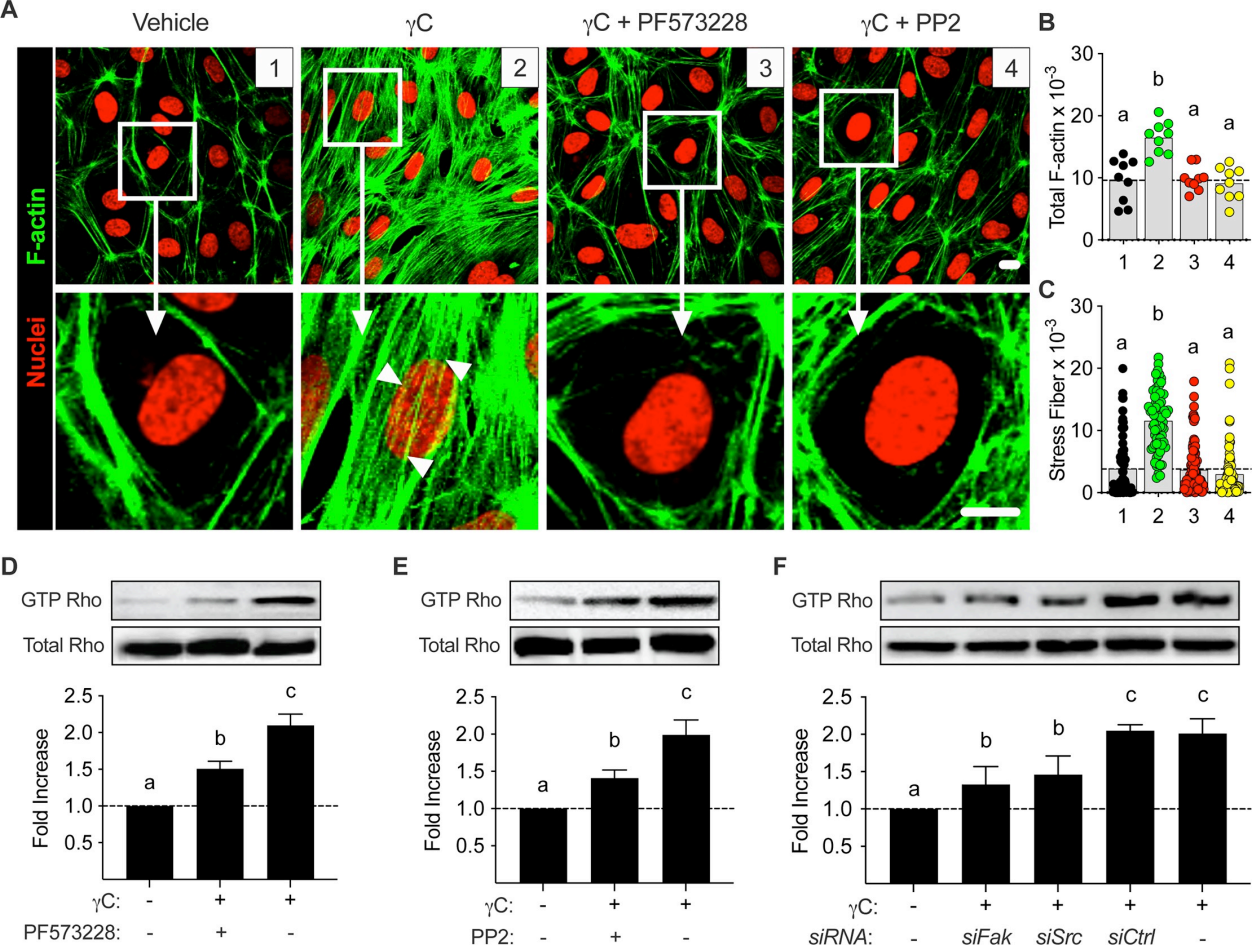
FAK and Src inhibition reduces γC-induced RhoA activity and stress fiber formation. RLMVECs were grown to confluence on glass bottom microwells and pre-treated with vehicle control, FAK (PF573228, 1μM), or Src inhibitor (PP2, 10μM), followed by γC (10 μg/mL) treatment for 10 minutes prior to F-actin labeling with AF 647-conjugated phalloidin. Representative images obtained using confocal microscopy (A). Arrows show heavy areas of γC-induced stress fiber formation and reduction of stress fibers by FAK and Src inhibition. B,C) Quantification of total F-actin intensity per micrograph (B) and stress fiber formation per cell (C). Data displayed as scatter dot plots with bars indicating the mean. RLMVECs were grown to confluence and pre-treated with (D) FAK inhibitor (PF573228, 1μM), or (E) Src inhibitor (PP2, 10μM) prior to γC (10 μg/mL) treatment for 10 minutes. Cells were lysed and analyzed for active RhoA (GTP Rho) (n = 4). F) RLMVECs were grown to 70% confluency, transfected with control, FAK, or Src siRNA, and then treated 48 hours post-transfection with γC (10 μg/mL) or vehicle control for 10 minutes followed by pull-down assay and immunoblotting for the detection of active RhoA (GTP Rho). siRNA knockdown of FAK and Src reduced γC-induced Rho activation by approximately 50% when compared to control siRNA. Blots and average optical densities are representative of three independent experiments, (n = 3). Groups with the same letter are not significant from each other (p > 0.05).

Our final experimental finding deduced the relationship between γC and GTP-Rho. To investigate whether FAK and/or Src activation influences cytoskeleton arrangement, F-actin morphology was revealed by AF-647 conjugated phalloidin staining and confocal microscopy (Fig 7A–7C). γC (10 μg/mL) treatment heightened F-actin stress fiber formation with dense and rough outlines in the cytoplasm and perinuclear regions of RLMVECs. Pharmacological inhibition of FAK and Src, using PF573228 (1μM) and PP2 (10μM) respectively, reduced γC-induced stress fiber formation (Fig 7A–7C). Active RhoA, GTP-RhoA, is known to induce cytoskeleton rearrangement [36, 37], and since FAK and Src are implicated in cell contractility mechanisms regulated by RhoA [21, 38], we tested whether inhibition of FAK and/or Src effected γC-induced RhoA activation. Using a RhoA GTPase pull-down assay as an indicator of RhoA activation, in cultured RLMVECs we demonstrated that γC induced activation of RhoA was attenuated by FAK or Src inhibition (Fig 7D–7F). To further elucidate the role of FAK and Src, we showed that siRNA knockdown of FAK and Src independently reduced γC-mediated activation of GTP-Rho (Fig 7D). All blot analysis showed no noticeable difference in total Rho expression.

## Discussion

Although the roles of FAK and Src have been implicated in endothelial barrier dysfunction caused by various inflammatory mediators [10, 20, 26, 39], their specific effects in FDP-γC-induced vascular leakage have not been reported. Within this study we demonstrate 1) γC induces microvascular hyperpermeability *in vivo* and endothelial barrier dysfunction in cultured endothelial cells; 2) The activation of FAK and Src contribute to the endothelial barrier alteration in response to γC; 3) FAK activates Src through an increase of phosphorylation at residue Y416; 4) γC-induced FAK and/or Src activity promotes AJ dissociation; and 5) FAK and/or Src regulate γC-induced RhoA activation and F-actin stress fiber formation. To the best of our knowledge, our study provides the first line of evidence demonstrating cellular signaling mechanisms involved in γC-induced microvessel hyperpermeability. Increase of circulating D-dimer, one of the major FDPs has been clinically associated with thrombotic disorders and systemic inflammation [4, 40]. Whether more specific biomarkers, such as γC can be used as a predictor of trauma severity warrants further investigation. In our study, the effect of γC on permeability in the animal model produced a slower initial response but a longer duration in comparison to the effect in cultured endothelial cells. The observation was consistent with our previously published data [5]. The longer time course of hyperpermeability *in vivo* is likely contributed by the complexity of vascular tissue structure and the impact of blood circulation including delayed contact between γC and endothelium, involvement of glycocalyx and participation of plasma proteins, blood cells that could produce a longer hyperpermeability effect. We further complement cell culture techniques with animal models to provide direct evidence for the involvement of FAK/Src signaling in endothelial barrier hyperpermeability caused by γC. Moreover, immunoprecipitation assay, immunofluorescence labeling, and immunoblotting demonstrate that γC-mediated FAK and Src activation is capable of catalyzing downstream pathways leading to AJ disassembly, and endothelial cytoskeleton contractibility to induce endothelial barrier dysfunction. Currently, some FAK and Src inhibitors have been tested to examine their roles in cell migration and angiogenesis for the beneficial effect in cancer treatment [33, 41]. In our study, we provide a new perspective of these inhibitors, which has the potential to rescue endothelial barrier dysfunction in post severe trauma.

FAK is a well-established key-regulator of Src and RhoA pathways, the activation of which lead to cell-cell junction loosening, cytoskeleton contractility, and vascular hyperpermeability [12, 20, 34]. Several lines of evidence have demonstrated that inhibition of FAK effectively blocks endothelial barrier dysfunction [7, 13, 14]. For instance, it was reported that blocking FAK activation by FRNK (FAK-related non-kinase) reduced activated neutrophil-induced vascular leakage [13]. FAK inhibitor (PF573228) has been shown to attenuate AGES-induced and Tys (analog of sphingosine 1-phosphate)-induced TER elevation [16, 35]. While both FAK and Src independently initiate distinct pathways, FAK is responsible for Src activation through FAK autophosphorylation on Y397, which creates a binding site for SH2 domain of Src, allowing Src to autophosphorylate residue Y416 [7, 34, 42]. FAK mediates the association of Src with focal adhesions using the SH2 domain [34]. FAK inhibitor Y15 reduced Src phosphorylation in colon cancer cells in a dose-dependent manner [43]. Consistent with these reports, our current data reports that FAK inhibition or genetic knockdown diminishes γC-induced Src phosphorylation, therefore suggesting that FAK is upstream of Src.

Our study also supports the well-established link that Src plays in endothelial barrier function [11, 20, 26, 27]. Src remains in an inactive state through association with the SH2 domain and Y527 [44]. Upon activation, Y527 dephosphorylates causing destabilization of the SH2, SH3 and other kinase domains, and Y416 autophosphorylation [7, 44]. γC-induced barrier leakage involves phosphorylation of Src Y416, a critical phosphorylation site also known to be responsible for mediating thrombin barrier dysfunction [45]. Activated neutrophils also stimulate Src activity through phosphorylation of residue Y416, thus mediating barrier dysfunction in human endothelial cells and hyperpermeability in porcine coronary venules [20]. While our data supported that γC increases tyr416 phosphorylation of Src, an event known to upregulate Src activity by using a phospho-specific antibody against Src, the same antibody also recognizes similar phosphorylated sites in other Src family kinases such as Yes, Fyn and Lyn [46, 47]. Therefore, our data do not exclude the potential effect on the endothelial barrier function by γC through other Src family kinases. Further experimentation revealed that Src inhibitor PP2 attenuated γC-induced albumin hyperpermeability in rat mesenteric microvessels and attenuated drop in TER in cultured monolayers. These data supported other studies that demonstrate improved endothelial barrier function upon treatment with PP2 [16, 27, 33]. Src inhibitors protect monolayers from increased permeability through regulation of AJ proteins [16, 19, 45]. Junctional protein regulation can be triggered by Src mediated intracellular signaling events [48]. We show that γC-mediated VE-Cadherin phosphorylation in a Src-dependent manner, supporting the established role that Src serves as a key element regulating junctional disorganization in response to many inflammatory mediators.

The integrity of the endothelial barrier is largely dependent on AJ at cell-cell contact and many inflammatory mediators promote vascular hyperpermeability through AJ signaling pathways [20, 31, 36]. Our results indicate that γC promotes phosphorylation of VE-Cadherin at Y658 in a dose-dependent manner, followed by subsequent barrier dysfunction through AJs dissociation, which are regulated by FAK and Src activation. Consistent with our findings, other groups have also reported that increase in VE-Cadherin phosphorylation leads to AJ opening and barrier dysfunction [19, 31 49]. Integrin antagonist cilengitide induces VE-Cadherin phosphorylation at Y658 and Src inhibition attenuates phosphorylation at this residue and reduces cilengitide-induced disappearance of VE-Cadherin at the cell-cell contacts [50]. Additional studies have supported the role of FAK-mediated AJ dissociation [8, 39]. In our study, siRNA knockdown or pharmacological inhibition of FAK or Src significantly improved AJ protein disorganization in response to γC and attenuated vascular hyperpermeability in γC-treated lung and mesenteric microvasculature. Downregulation of FAK by siRNA in cultured cells attenuated the increase of VE-Cadherin phosphorylation at Y658 by γC. FAK appears to modulate AJ activity by facilitating the transportation of c-Src to AJs, where Src induces VE-Cadherin phosphorylation [39]. We demonstrate that γC-induced FAK activity promotes Src phosphorylation at residue Y416. While in support of our findings, it has been reported that FAK is capable of regulating the activation of Src [18, 34, 42], others have shown the activation of FAK by Src [16, 17]. This suggests that the dynamic relationship between FAK and Src may vary based on the stimulus involved and the dynamic state of the endothelium. It is possible that the two tyrosine kinases interact through a feedback mechanism, since different residues require the activation of the other.

Over the past decades, RhoA pathway has been implicated in endothelial hyperpermeability [6, 9, 21, 25], and Rho-GTP pull down assays in our previous study supported γC promoting barrier dysfunction through RhoA activation and MLC phosphorylation [5]. RhoA is known to play a direct role in cytoskeletal actin remodeling and F-actin polymerization [6, 25, 36, 37] and formation of F-actin fibers can lead to cytoskeletal reorganization and cell contraction causing subsequent gap formation, which allows blood components (fluid and plasma protein) to leak into interstitial tissue [37]. Activated-neutrophils have been shown to increase endothelial permeability through RhoA pathway activation, stress fiber formation, and intercellular gap formation [24, 25]. Our results indicate that γC elicits similar gap formation through F-actin reorganization and activation of the RhoA pathway. Evidence suggests a crosstalk between FAK/Src signaling and RhoA during paracellular permeability [21, 38, 51]. Src phosphorylation is necessary for functional formation of integrin-dependent focal adhesion attachment to actin stress fibers in fibroblasts [52], and FAK induces RhoA phosphorylation leading to gap formation and endothelial barrier dysfunction [51, 53]. We show that either FAK or Src inhibition can independently reduce F-actin stress fiber formation and significantly attenuate Rho GTP activity induced by γC.

In summary, we have shown that FAK and Src are implicated in the hyperpermeability response to γC. Inhibition of both FAK and Src attenuates γC-mediated Rho activation, AJ dissociation, F-actin polymerization, and subsequent endothelial barrier dysfunction and microvascular hyperpermeability, where FAK is an upstream regulator of Src. Our data provide experimental evidence supporting the roles of FAK and Src in mediating microvascular hyperpermeability elicited by γC.

## Supporting information

S1 Table Cells and reagents. (DOCX)
