# Supplementary material for: Focal adhesion kinase and Src mediate microvascular hyperpermeability caused by fibrinogen- γC- terminal fragments
Source: PLoS One. 2020 Apr 30;15(4):e0231739. doi: 10.1371/journal.pone.0231739 (PMC7192500; doi:10.1371/journal.pone.0231739)
Supplement: S1 Table — (DOCX) [file pone.0231739.s001.docx]

| **Supplementary Table I - Reagents** | |
| --- | --- |
| **Reagent/Drug** | **Company** |
| Albumin-Fluorescein Isothiocyanate Conjugate | Sigma (St. Louis, MO) |
| Basic Endothelial Nucleofector Solution | Amaxa™ Biosystems |
| Chambered Glass Microwells | Mat Tek (Ashland, MA) |
| ECIS Assays | Applied Biophysics (Troy, NY) |
| Endothelial Basal Medium (EBM) | Lonza (Walkersville, MD) |
| FAK Polyclonal Primary Antibody | Cell Signaling (Beverly, MA) |
| *FAK siRNA(1) - M-090463-01-0005 | Dharmacon inc. (Lafayette, CO) |
| *FAK siRNA(2) - sc-156037 | Santa Cruz Biotechnology (Santa Cruz, CA) |
| Anti-VE-cadherin C-19; sc6458 | Santa Cruz Biotechnology (Santa Cruz, CA) |
| Anti-Beta-catenin H-102; sc7199 | Santa Cruz Biotechnology (Santa Cruz, CA) |
| MCDB-131 Complete Medium | VEC technologies (Rensselaer, NY) |
| PF573228 Inhibitor | Tocris (Minneapolis, MN) |
| pFAK (Y397) Polyclonal Primary Antibody | Cell Signaling (Beverly, MA) |
| PP2 Inhibitor | Tocris (Minneapolis, MN) |
| pSrc (Y416) Polyclonal Primary antibody | Cell Signaling (Beverly, MA) |
| Rat Lung Microvascular Endothelial Cells | VEC technologies (Rensselaer, NY) |
| Rho Monoclonal Primary Antibody (clone 55) | Upstate (Lake Placid, NY) |
| GTP RhoA Pulldown Assay Kit | Upstate (Lake Placid, NY) |
| RhoA Monoclonal Primary Antibody | Santa Cruz Biotechnology (Santa Cruz, CA) |
| Scrambled siRNA | Santa Cruz Biotechnology (Santa Cruz, CA) |
| *Src siRNA(1) sc-270199 | Santa Cruz Biotechnology (Santa Cruz, CA) |
| *Src siRNA(2) sc-270126 | Santa Cruz Biotechnology (Santa Cruz, CA) |
| ß-actin Monoclonal Primary Antibody | Santa Cruz Biotechnology (Santa Cruz, CA) |
| Vectasheild Mounting Medium with DAPI | Vector laboratories (Burlington, CA) |

**Supplementary Materials and Methods**

**Silencing RNA Sequences**

To achieve siRNA-mediated gene knockdown commercially available pools of siRNA wet-lab validated to target rat *FAK* (NM_013081) and *Src* (NM_031977). All sequences are provided in the 5’ - 3’ direction.

| *Sequences of siFAK(1) pool* (*M-090463-01-0005)*: | | | |
| --- | --- | --- | --- |
|  | D-090463-01: | Sense: GUCCAACUAUGAAGUGUUA | Antisense: UAACACUUCAUAGUUGGAC |
|  | D-090463-02: | Sense: GGGUCAAGUUGGAUUAUUU | Antisense: AAAUAAUCCAACUUGACCC |
|  | D-090463-03: | Sense: ACACCAAGUUCGAGUACUA | Antisense: UAGUACUCGAACUUGGUGU |
|  | D-090463-04: | Sense: CUGCUUAUCUUGACCCAAA | Antisense: UUUGGGUCAAGAUAAGCAG |
| *Sequences of siFAK(2) pool (sc-156037):*  sc-156037A: Sense: GGAAGACAGUACUUACUAU Antisense: AUAGUAAGUACUGUCUUCC  sc-156037B: Sense: GUGAUCGGUCGAAUUGAAA Antisense: UUUCAAUUCGACCGAUCAC  sc-156037C: Sense: GGAAGAGCGAUUCCUGAAA Antisense: UUUCAGGAAUCGCUCUUCC  *Sequences of siSrc(1) pool (sc-270199):* | | | |
|  | sc-270199A: | Sense: GAUCACUAGACGGGAAUCA | Antisense: UGAUUCCCGUCUAGUGAUC |
|  | sc-270199B: | Sense: GCUUACUACUCCAAACAUG | Antisense: CAUGUUUGGAGUAGUAAGC |
| *Sequences of siSrc(2) pool (sc-270126):* | | | |
|  | sc-270126A: | Sense: CCCAUCCUAUAGAUUCAUA | Antisense: UAUGAAUCUAUAGGAUGGG |
|  | sc-270126B: | Sense: GGAUGACGUAAAGGUCAAA | Antisense: UUUGACCUUUACGUCAUCC |
|  | sc-270126C: | Sense: CUGUGACGGCGAUAAAUCA | Antisense: UGAUUUAUCGCCGUCACAG |
